# Development and testing of a Shared decision making ExpeRience in mENtal hEalth (SERENE) measure

**DOI:** 10.1192/j.eurpsy.2023.1916

**Published:** 2023-07-19

**Authors:** M. Chmielowska

**Affiliations:** Clinical, Educational and Health Psychology, UCL, London, United Kingdom

## Abstract

**Introduction:**

Shared decision making (SDM) is a health communication approach focusing on patient-clinician interactions around treatment decisions, with the goals of improving clinical and functional outcomes and providing personalized care. Moreover, decision making may need to be negotiated between, and communicated to, multiple health and social care practitioners, as well as patients and their social networks (SNs). The skills for sharing and discussing personal information with patients, and their SNs, can be hard to embed in mental health services. Compared to physical health, SDM in mental health is characterized by inconsistent definitions, models, and measurement, and evidence for the effectiveness of SDM interventions is inconclusive. Therefore, there is a need to define what is considered an effective SDM approach in mental healthcare, and to determine the core elements and steps required for its successful implementation in mental health populations.

**Objectives:**

To better understand the concept and role of patients’ social networks in treatment decision-making in mental health.

**Methods:**

A two-phase process (compliant with the International Patient Decision Aid Standards) will be used to develop the first polyadic SDM measure of patient experience to support a wide range of models of mental health care. The new measure of patient experience will focus for the first time on the patient’s perception of individual qualities/behaviour of a patient, clinician, caregiver, and their interaction. Phase I of this thesis involved reviewing the existing SDM interventions and SN interventions in mental health, as well as reviewing the existing instruments used to measure SDM in mental health research and practice. Phase II will involve developing and revising the new measure of SDM using stakeholder feedback in an iterative process. Stakeholders will include patients, their caregivers, and clinicians recruited from community mental health services in England.

**Results:**

To define SDM and the most relevant aspects of it, data from the literature review and exploratory focus groups will be evaluated together. A pool of draft questions will be generated, covering all aspects of the identified themes. These questions will be further refined following discussions with people with lived experience of mental illness (i.e., peers) and academics who have the relevant expertise in SDM and psychometric measure development. This will establish content validity and ensure wording clarity, to be accessible for all English language requirements.

**Conclusions:**

The new SDM measure will ensure effective evaluation of SDM experiences of people with mental illness. It will be an important step forward in advancing the study and application of SDM in mental health care. It will lay the foundation for further research into the needs of patients and their SNs regarding SDM in mental healthcare.

**Disclosure of Interest:**

None Declared

